# Enhancing grant-writing expertise in BUILD institutions: Building infrastructure leading to diversity

**DOI:** 10.1371/journal.pone.0274100

**Published:** 2022-09-22

**Authors:** Robert A. Hiatt, Yazmin P. Carrasco, Alan L. Paciorek, Lauren Kaplan, Marc B. Cox, Carlos J. Crespo, Andrew Feig, Karsten Hueffer, Harris McFerrin, Keith Norris, Elizabeth Roberts-Kirchhoff, Carrie L. Saetermoe, Gillian Beth Silver, Katherine Snyder, Arturo R. Zavala, Audrey G. Parangan-Smith, on behalf of the Diversity Program Consortium

**Affiliations:** 1 Department of Epidemiology and Biostatistics and Helen Diller Family Comprehensive Cancer Center, University of California San Francisco, San Francisco, CA, United States of America; 2 Department of Epidemiology and Biostatistics, University of California San Francisco, San Francisco, CA, United States of America; 3 Center for Vulnerable Populations, Zuckerberg San Francisco General Hospital and Trauma Center, University of California, San Francisco, San Francisco, CA, United States of America; 4 Department of Pharmaceutical Sciences, Department of Biological Sciences, and Border Biomedical Research Center, University of Texas at El Paso, El Paso, TX, United States of America; 5 Oregon Health and Science University and Portland State University Joint School of Public Health, Portland, OR, United States of America; 6 Department of Chemistry, Wayne State University, Detroit, MI, United States of America; 7 Department of Veterinary Medicine, University of Alaska Fairbanks, Fairbanks, AK, United States of America; 8 Biology Department, Xavier University, New Orleans, LA, United States of America; 9 Department of Medicine, David Geffen School of Medicine at UCLA, Los Angeles, CA, United States of America; 10 Department of Chemistry and Biochemistry, University of Detroit Mercy, Detroit, MI, United States of America; 11 Department of Psychology, California State University Northridge, Northridge, CA, United States of America; 12 ASCEND Center for Biomedical Research, Division of Research & Economic Development, Morgan State University, Baltimore, MD, United States of America; 13 Department of Mathematics and Computer Science, University of Detroit Mercy, Detroit, MI, United States of America; 14 Department of Psychology, California State University, Long Beach, Long Beach, CA, United States of America; 15 Department of Biology, San Francisco State University, San Francisco, CA, United States of America; University of Michigan, UNITED STATES

## Abstract

**Background:**

The lack of race/ethnic and gender diversity in grants funded by the National Institutes of Health (NIH) is a persistent challenge related to career advancement and the quality and relevance of health research. We describe pilot programs at nine institutions supported by the NIH-sponsored Building Infrastructure Leading to Diversity (BUILD) program aimed at increasing diversity in biomedical research.

**Methods:**

We collected data from the 2016–2017 Higher Education Research Institute survey of faculty and NIH progress reports for the first four years of the program (2015–2018). We then conducted descriptive analyses of data from the nine BUILD institutions that had collected data and evaluated which activities were associated with research productivity. We used Poisson regression and rate ratios of the numbers of BUILD pilots funded, students included, abstracts, presentations, publications, and submitted and funded grant proposals.

**Results:**

Teaching workshops were associated with more abstracts (RR 4.04, 95% CI 2.21–8.09). Workshops on grant writing were associated with more publications (RR 2.64, 95% CI 1.64–4.34) and marginally with marginally more presentations. Incentives to develop courses were associated with more abstracts published (RR 4.33, 95% CI 2.56–7.75). Workshops on research skills and other incentives were not associated with any positive effects.

**Conclusions:**

Pilot interventions show promise in supporting diversity in NIH-level research. Longitudinal modeling that considers time lags in career development in moving from project development to grants submissions can provide more direction for future diversity pilot interventions.

## Introduction

Diversity in racial, ethnic, and cultural backgrounds in health-related research can improve research quality and relevance to communities and advance both research innovation and career development of those underrepresented in NIH-funded work. The low rates of proposals submitted to the National Institutes of Health (NIH) by faculty from underrepresented groups reflect the lack of diversity in the medical research community [1–3]. Diverse NIH-funded researchers can attract students from underrepresented backgrounds, creating opportunities for a robust pipeline of diverse NIH grant applicants and opening doors for those underrepresented in health research [4–7].

In 2012, recommendations to the NIH to develop and support diversity in research resulted in initiatives to address this challenge including the Diversity Program Consortium (DPC) and its two components, the Building Infrastructure Leading to Diversity (BUILD) program [5, 8] and the National Research Mentoring Network (NRMN) [6]. The BUILD initiative was subsequently funded at ten teaching-intensive institutions that educate high proportions of underrepresented and low-income students in widely distributed locations across the United States (see below and S1 Appendix). The ten BUILD sites are heterogeneous in their academic structures (e.g. teaching vs tier 1 research institutions), demographics of students and their faculty, and their partnership relations with pipeline institutions and research-intensive universities [9]. All BUILD sites aim to increase mentorship and training for faculty to develop their research skills to promote a diverse research environment more representative of the US population [10].

The BUILD programs provided mini-grant or pilot funding to faculty to develop research projects and collect preliminary data for writing and submitting grant applications, an essential skill in research careers based on competitive NIH funding that many faculty may lack [6]. As part of this effort, training in and support for grant writing were provided to the faculty [11]. Keeping pace with frequent changes in procedures at federal grant agencies requires ongoing faculty support in grant writing and research administration. Therefore, administrative workshops were offered as part of some programs.

In this study, we describe examples of local in-house pilot programs at nine BUILD institutions, which provided funding to faculty and opportunities for mentorship of students. The 10^th^ institution did not have a pilot program thus did not submit data for this evaluation. No non-BUILD programs participated or received training as part of these awards. The goal was to increase faculty competitiveness for securing external funding and promote successful and sustainable research careers. BUILD also aimed to provide students with undergraduate research opportunities and mentoring. These pilot programs address one of the aims of the BUILD program to “enhance faculty mentoring and research skills” [9]. To inform future research enhancement programs, we: 1) provide a description of pilot programs BUILD institutions deployed for their faculty; 2) examine the utilization and associated outcomes of these initiatives; and 3) discuss lessons learned. Each institution structured the program to fit their own context, and no centralized effort was made to standardize the approach. We examined both the underlying institutional capacity-building and the productivity outcomes of faculty (and student involvement) in research activities as measured by conference presentations, publications, submitted proposals and successful funding in the first four years of Phase 1 of the BUILD program [9].

## Methods

### Defining program characteristics

The BUILD awards differ from other NIH-funded training grants in that they aim to impact simultaneously students, faculty, and institutions by implementing a variety of interventions targeted toward research capacity-building, mentorship, and institutional change. In 2014, ten competitive 5-year BUILD awards were issued to undergraduate institutions across the country. Eligibility for BUILD primary institutions included having less than $7.5 million in total NIH research project grant funding and a student population with at least 25 percent Pell Grant recipients. The funded BUILD institutions serve geographically, socioeconomic, and racially diverse populations, and include historically Black colleges and universities, Hispanic-serving institutions, Asian American/Native American/Alaska Native/Pacific Islander-serving institutions, and institutions that target outreach to special populations. Higher Education Research Institute (HERI) surveys of incoming BUILD students from 2015–2019 documented that many BUILD scholars were learners from groups historically underrepresented in biomedical sciences [9]. Funded BUILD institutions partner with nearly 100 other institutions (S1 Appendix), some of which had basic science education programs and that were research-intensive, to broaden the opportunities for the students participating in biomedical research training and maximize opportunities for faculty and staff development. The ten funded BUILD institutions with links to their programs are:

- California State University, Long Beach: CSULB BUILD
- California State University, Northridge (CSUN): (Promoting Opportunities for Diversity and Education and Research) BUILD PODER
- Morgan State University (MSU): (A Student-Centered, Entrepreneurship Development Training Model to Increase Diversity in the Biomedical Research Workforce) ASCEND
- Portland State University (PSU): (Enhancing Cross-Disciplinary Infrastructure Training at Oregon) BUILD EXITO
- San Francisco State University: Enabling Students to Represent in Science SF BUILD
- University of Alaska, Fairbanks (UAF): (Biomedical Learning and Student Training) BUILD BLaST
- University of Detroit Mercy (UDM): (Research Enrichment Building Infrastructure Leading to Diversity) ReBUILDetroit
- University of Maryland, Baltimore County: (UMBC) STEM BUILD@UMBC
- University of Texas at El Paso (UTEP): (Southwest Consortium of Health-Oriented Education Leaders and Research Scholars) BUILDing SCHOLARS
- Xavier University of Louisiana: BUILD Project Pathways

**Fig 1:**
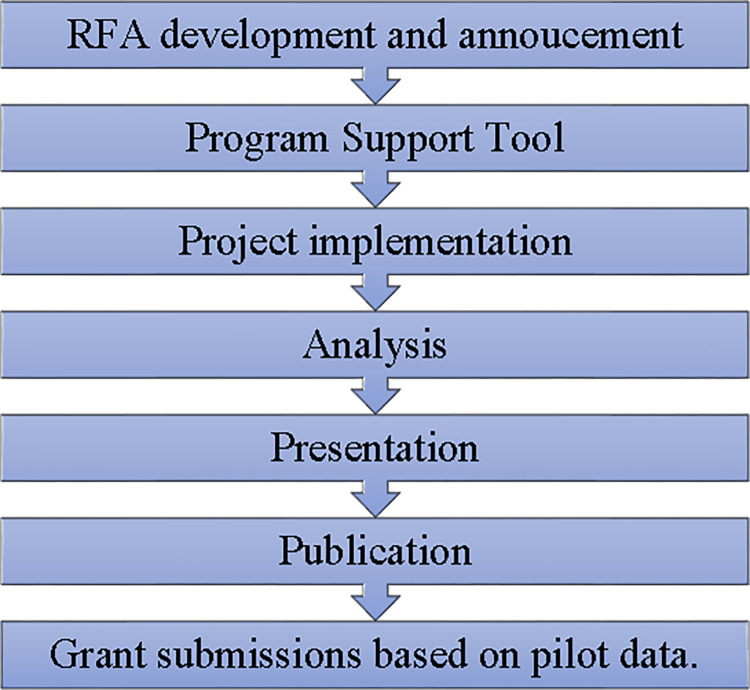
Pilot project program framework. Steps to grant submission.

All institutions except UMBC developed a pilot project program designed to support faculty in writing research grants while simultaneously providing an educational platform to promote the pursuit of research by underrepresented students. The approach taken by BUILD primary institutions followed the steps of pilot Request For Application (RFA) development and announcement, using program support tools, implementing the research, and measuring key outcomes (Fig 1).

Each BUILD primary institution had some program tools already in place, which provided background support for the development of their pilot project programs; details of their approaches vary slightly. It was not possible to separate out faculty use of the BUILD funded program tools from research infrastructure (e.g., Clinical and Translational Science Institutes, etc.) that already existed in the funded institutions because the baseline HERI survey did not differentiate between the two. We, therefore, treated all such tools as potentially supporting BUILD funded pilot projects. These ten tools included 1) workshops on teaching; 2) developing research skills, and grant writing; 3) funds for sabbaticals; 4) institutional funds for travel and research; 5) training for administrative leadership; 6) incentives for developing new courses; 7) incentives for integrating technology in the classroom; and 8) incentives to integrate culturally competent and student-centered practices in the classroom. Although details differ in implementation, all sites shared the aim of supporting faculty research productivity and each element that is reported here was used by at least some HERI faculty survey participants at every site. In our analysis we included an evaluation of the role of program support tools. Seven quantitative productivity outcomes of interest were measured at each institution as reported by participants associated with the BUILD pilot programs: 1) numbers of BUILD pilots funded, 2) students included, 3) abstracts, 4) presentations, 5) publications, 6) submitted grant proposals, and 7) funded grant proposals.

### Data collection and standard reporting to NIH

The BUILD Coordination and Evaluation Center (CEC) annually collected data directly from faculty as a component of the Consortium-Wide Evaluation Plan (CWEP). In 2016–2017 faculty data were gathered using the HERI Faculty Survey, which is administered triennially [1]. Survey participants included faculty participating in various BUILD interventions including the pilot project program and a comparison group of faculty at the same institution. The target was 50 responses per BUILD program: 25 participating in the BUILD program and 25 for the comparison group. However, some interventions targeted most or all faculty in a department, college, or institution, leaving few faculty without exposure to the interventions for comparison, so the intent to have an internal comparison group was not feasible.

The HERI Faculty survey is a self-assessment comprising 55 questions encapsulating individual demographics, teaching and research experience, current scope and scale of roles in teaching, research or administration, and attitudes and uses of program support tools [12]. The survey included validated instruments from HERI so data could be compared with other studies that also used these validated instruments. Table 1 summarizes aspects of sample selection for the 2016–2017 survey. Most programs administered the survey during Fall 2016; the UDM administered the survey in Winter 2017. Some programs offered raffled incentives, some offered incentives to departments with high response rates, and others offered no incentives. The Survey data we analyze here are only from faculty who participated in BUILD (Table 1).

**Table 1:** Sampling description for HERI faculty survey 2016–2017 by BUILD institution.

Institution	Departments	Sampling
CSULB [13]	Faculty from biomedical and health-related departments, including tenure track faculty as well as instructors and lecturers.	All faculty invited, with plans for CEC to sample after administration. (Subsequently the decision was made to include all respondents.)
CSUN [14]	Full-time faculty from biomedical departments	Initially invited a selected sample, but low response prompted an expansion to all involved with the BUILD program
MSU [15]	All biomedical faculty, including gerontology, consumer science, and architecture; Adjunct faculty included.	No sampling
PSU [16]	All biomedical faculty, including social work; research and teaching faculty with at least half-time appointments	No sampling
UDM Detroit Mercy [17]	Institutionally administered survey to all faculty	Selected biomedical faculty after survey
SFSU [18]	Faculty from biomedical departments	No sampling, but some faculty removed as part of pre-survey opt-out process
UAF [19]	Faculty from biomedical departments at Fairbanks campus and remote sites	CEC selected sample invited
UMBC [20]	Faculty from biomedical departments	No sampling
UTEP [21]	Faculty from biomedical departments; included non-tenure track faculty	CEC selected sample invited
Xavier [22]	Faculty from biomedical departments, including the Division of Basic and Pharmaceutical Sciences in the College of Pharmacy	No sampling

CSULB = California State University, Long Beach; CSUN = California State University, Northridge; MSU = Morgan State University; PSU = Portland State University; SFSU = San Francisco State University; UAF = University of Alaska, Fairbanks; UDM/WSU = University of Detroit, Mercy/Wayne State University; UMBC = University of Maryland, Baltimore County; UTEP = University of Texas at El Paso; Xavier = Xavier University of Louisiana.

The nine BUILD primary institutions, excluding the UMBC, collected data from faculty participants of the pilot project program on productivity as part of their annual progress report to the NIH. We collected and analyzed the productivity data from progress reports submitted from 2015–2018 that summarized data per institution; counts of BUILD pilots funded, students included, abstracts, presentations, publications, grant proposals submitted, and grant proposals funded.

To describe the ten program support tools (not to be confused with the ten BUILD sites), we calculated the proportion of participants who used each tool at each institution. We characterized institutions by the distributions of gender, race/ethnicity, and faculty experience with education.

To assess which program support tools might be aligned with which productivity measures, we first designated each institution as a binary high or low use of the tool which, depending on how commonly the tool was used overall, was based on a cutoff of at least one half, one quarter, or one tenth of faculty. We then fit a Poisson regression model to the productivity count data and tool use data at the nine institutions in the study. The standard error of the regression coefficient was corrected for overdispersion by using the dispersion parameter, and the regression coefficient was exponentiated to present the association as a rate ratio of an increase in productivity for institutions that had high use of a program support tool compared to low. We used the Bonferroni adjustment to the 77 association tests to be conservative in concluding that any tool was significantly associated with any productivity measure. All tests were two-sided and considered statistically significant at the Type I error rate below 0.00065. Analyses were done using SAS software, Version 9.4, SAS Institute Inc. Data will be made available upon request.

## Results

### Participant characteristics

A total of 450 individuals participated in the 2016–17 HERI Faculty Survey; 51 participants were excluded from the analysis since no productivity data was available from the participants’ institution (i.e., UMBC). As shown (Table 2), 220 (55%) participants identified as women, and 82 (20%) belong to racial/ethnic minority groups. Of the 313 respondents who reported race/ethnicity, 33 (11%) were Black, 40 (13%) Asian, 19 (6%) Latinx, 18 (6%) multiracial, 12 (4%) other races, and 181 (58%) White. Median age was 48. The largest academic group was Assistant Professors (152; 38%) with a close to equal distribution of Associate Professors (109; 28%) and Professors (104; 26%); out of those faculty 204 (52%) were tenured. Participants had a median of 11 years of teaching experience, teaching two courses per semester, mentoring four research students per years, and spending seven hours per week on research and scholarly writing.

**Table 2:** Survey characteristics of sites. HERI survey, 2016–17.

	UAF	CSULB	CSUN	SFSU	Xavier	MSU	UDM	PSU	UTEP	Total
Total N (%)	26	82	24	36	63	34	27	60	47	399
**Rank**										
**Professor**	7 (27%)	31 (38%)	7 (29%)	15 (42%)	11 (17%)	2 (6%)	2 (37%)	10 (17%)	11 (23%)	104
**Associate Professor**	7 (27%)	21 (26%)	8 (33%)	9 (25%)	20 (32%)	11 (32%)	9 (33%)	14 (23%)	10 (21%)	109
**Assistant Professor**	12 (46%)	30 (37%)	9 (38%)	10 (28%)	29 (46%)	16 (47%)	6 (22%)	25 (42%)	15 (32%)	152
**Lecturer**	0	0	0	1 (3%)	2 (3%)	5 (15%)	1 (4%)	2 (3%)	5 (11%)	16
**Instructor**	0	0	0	0	1 (2%)	0	1 (4%)	8 (13%)	5 (11%)	15
**Unknown**	0	0	0	1 (3%)	0	0	0	1 (2%)	1 (2%)	3
**Tenured**	14 (54%)	52 (63%)	10 (42%)	23 (64%)	31 (49%)	13 (38%)	19 (70%)	21 (35%)	21 (45%)	204
**Female**	14 (54%)	49 (60%)	16 (67%)	20 (56%)	32 (51%)	19 (55%)	16 (59%)	30 (50%)	24 (51%)	220
**Partnered**	14 (54%)	45 (55%)	13 (54%)	23 (64%)	37 (59%)	14 (41%)	23 (85%)	36 (60%)	31 (66%)	236
**Race**										
**White**	9 (35%)	44 (54%)	8 (33%)	16 (44%)	25 (40%)	4 (12%)	20 (74%)	34 (57%)	21 (45%)	181
**Black**	0	1 (1%)	2 (8%)	3 (8%)	13 (21%)	9 (26%)	3 (11%)	1 (2%)	1 (2%)	33
**E Asian**	1 (4%)	9 (11%)	1 (4%)	3 (8%)	1 (2%)	1 (3%)	1 (4%)	3 (5%)	2 (4%)	22
**S Asian**	1 (4%)	0	1 (4%)	0	3 (5%)	4 (12%)	0	1 (2%)	1 (2%)	11
**Other Asian**	0	2 (2%)	1 (4%)	2 (6%)	1 (2%)	0	0	0	0	6
**Multiple Asian**	0	0	1 (4%)	0	0	0	0	0	0	1
**Mexican**	1 (4%)	2 (2%)	0	3 (8%)	0	0	0	0	7 (15%)	13
**Other Latinx**	1 (4%)	1 (1%)	2 (8%)	0	0	0	0	1 (2%)	1 (2%)	6
**Other**	0	3 (4%)	0	1 (3%)	3 (5%)	2 (6%)	1 (4%)	0	2 (4%)	12
**Multiple races**	1 (4%)	5 (6%)	0	2 (6%)	2 (3%)	1 (3%)	0	3 (5%)	4 (9%)	18
**Unknown**	12 (46%)	15 (18%)	8 (33%)	6 (17%)	15 (24%)	13 (38%)	2 (7%)	17 (28%)	8 (17%)	96
**Hispanic**	2 (8%)	3 (4%)	2 (8%)	3 (8%)	0	0	0	1 (2%)	8 (17%)	19
**Not native English speaker**	3 (12%)	16 (20%)	4 (17%)	4 (11%)	5 (8%)	5 (15%)	2 (7%)	6 (10%)	11 (23%)	56
**Age median [range]**	52 [34,65]	48 [29,76]	46 [34,55]	48 [32,70]	49 [31,81]	46 [32,75]	52 [37,67]	52 [35,69]	47 [32,67]	48
**Years teaching; median [range]**	15 [1,32]	12 [1,44]	7 [1,20]	15 [2,41]	11 [1,47]	10 [1,50]	13 [0,41]	9 [1,47]	11 [2,49]	11
**Courses; median [range]**	1 [0,6]	3 [0,4]	3 [0,4]	2 [0,9]	3 [0,9]	3 [1,5]	3 [2,5]	2 [0,7]	2 [0,10]	2
**Students; median [range]**	3 [0,105]	5 [0,105]	8 [0,105]	3 [0,85]	3 [0,105]	3 [0,85]	15 [0,85]	2 [0,105]	5 [0,105]	4
**Hours research; median [range]**	15 [0,23]	7 [3,23]	11 [0,19]	11 [0,23]	3 [0,23]	5 [0,19]	3 [0,23]	11 [0,23]	11 [0,23]	7
**Salary monthly x $1000 median range]**	8 [6.5,14.6]	8.5 [6.3,11.3]	7.9 [5.4,10.6]	8.5 [6.5,11.5]	6.5 [4.6,11.5]	7.5 [4.5,11.3]	7.5 [3.8,9.5]	7.5 [3.8,14.6]	7.5 [3.8,14.6]	7.5
**Publications; median [range]**	5 [0,21]	4 [0,21]	3 [0,15]	3 [0,21]	2 [0,11]	2 [0,15]	3 [0,15]	4 [0,21]	5 [0,21]	3

### Use of program tools

The most used tools were workshops on teaching (59%), workshops on research skills (37%), workshops on grant writing (29%), institutional funds for travel (55%), grant funds from the institution (51%), incentives for technology (36%), and incentives for integrating culturally competent practices in the classroom (40%) (Table 3).

**Table 3:** The count and proportion of participants at each BUILD site who used a research tool at their institution, HERI Survey 2016–2017.

	UAF	CSULB	CSUN	SFSU	Xavier	MSU	UDM	PSU	UTEP	Total
**Workshop on teaching**	9 (47%)	38 (54%)	15 (75%)	14 (45%)	43 (80%)	15 (65%)	20 (77%)	16 (32%)	27 (69%)	197 (59%)
**Workshop on research skills**	4 (22%)	24 (36%)	8 (40%)	6 (19%)	23 (45%)	16 (67%)	14 (56%)	8 (17%)	17 (44%)	120 (37%)
**Workshop on grant writing**	4 (22%)	22 (30%)	8 (42%)	7 (23%)	11 (22%)	10 (43%)	10 (40%)	12 (24%)	9 (24%)	93 (29%)
**Sabbatical**	1 (6%)	11 (18%)	3 (21%)	4 (17%)	3 (9%)	0	3 (13%)	1 (3%)	0	26 (11%)
**Travel funds from institution**	7 (37%)	35 (51%)	16 (89%)	13 (42%)	25 (49%)	17 (65%)	17 (68%)	27 (53%)	20 (59%)	177 (55%)
**Research grants from institution**	8 (44%)	48 (67%)	12 (57%)	7 (24%)	19 (37%)	13 (52%)	18 (69%)	24 (48%)	18 (47%)	167 (51%)
**Training on Administrative Leadership**	5 (28%)	9 (14%)	3 (19%)	4 (14%)	4 (9%)	3 (14%)	2 (10%)	4 (9%)	4 (12%)	38 (13%)
**Incentive to develop new course**	4 (24%)	15 (21%)	5 (26%)	4 (13%)	14 (27%)	7 (33%)	11 (46%)	11 (24%)	12 (38%)	83 (27%)
**Incentive to integrate technology in classroom**	6 (35%)	21 (30%)	9 (43%)	7 (23%)	17 (35%)	11 (55%)	9 (39%)	9 (20%)	21 (64%)	110 (36%)
**Incentive to integrate culturally competent practices in the classroom**	4 (29%)	32 (45%)	12 (67%)	10 (33%)	16 (34%)	10 (50%)	11 (50%)	18 (38%)	8 (26%)	121 (40%)
**Average funding level for BUILD pilot projects**	$53147*	$14396	$50000	$25592	$40410	$59849¥	$25000	$73552	$20000	$38570

* Pilot project funding period ranged from 1–2 years

¥ Pilot project funding period ranged from 1–3 years

It is likely that some of these tools that faculty reported using were not newly developed for the BUILD program but are included because they characterize the institutional environment. Approximately three-quarters of participants reported using the teaching workshops at CSUN, Xavier, and UDM.

Approximately two-thirds of participants used workshops on research skills at MSU. At least one-half used institutional travel funds at CSULB, CSUN, MSU, UDM, PSU, and UTEP and, likewise, at least half used research grants provided by the institution at CSULB, CSUN, MSU, and UDM. At least one-half of participants used incentives for adding technology in the classroom at MSU and UTEP, and incentives for adding culturally competent practices in the classroom were used by at least one-half of participants used at MSU and UTEP and at CSUN, MSU, and UDM, respectively.

The pilot funding amount was approximately $10,000–50,000 per pilot and most were for a duration one or one and a half years, but others ranged up to five years (UAF 2–2.5 years; MSU 1–3 years (Table 3). The BUILD project grants amounts were on average at least $50,000 at four institutions: UAF, CSUN, MSU, and PSU. The average award amounts varied between institutions from $14,396 at CSULB to $73,552 at Portland State University.

### Productivity outcomes

The number of pilot projects supported by each BUILD program varied from five to 38 (Table 4). Most pilot programs were offered between 2015 and 2018; except for PSU where pilots were only supported in 2016–17. The average number of students engaged in the research supported by these projects overall was 42 with as many as 160 students involved at CSULB.

**Table 4:** Count of productivity measures at each BUILD primary institution, 2015–2018.

	UAF	CSULB	CSUN	SFSU	Xavier	MSU	UDM	PSU	UTEP	Ave
**Number of pilots funded**	25	38	9	9	19	20	11	10	5	16.2
**Students included**	30	160	0	48	72	22	23	13	9	41.9
**Abstracts**	1	5	0	6	69	11	6	5	6	12.1
**Presentations**	30	143	0	8	83	12	5	28	6	35.0
**Publications**	1	47	9	0	15	1	0	7	4	9.3
**Submitted grant proposals**	20	43	7	0	43	0	2	38	0	17.0
**Funded grant proposals**	10	13	NA	0	13	0	0	15	0	6.4

NA—Funded grant proposals not recorded for Northridge

An average of 12.1 abstracts, 35 presentations, and 9.3 publications per institution were produced in BUILD projects. CSULB reported the most peer-reviewed publications (47) generated from their pilot projects. Among all BUILD sites, an average of 17 grants were proposed and 6.4 successfully funded. Almost all of proposals (73%) were submitted to the NIH (N = 41) or other federal funders (e.g., National Science Foundation) (N = 49) and the number of grants submitted or funded from other sources was thus too small for meaningful comparison. The largest number of grants funded because of these BUILD pilot projects was 15 at PSU.

### Productivity outcomes and BUILD program tools

Often institutions where more faculty used a program tool there was lower productivity (Table 5). However, there were also various program tools that aligned with more productivity. At institutions where more faculty used a workshop on teaching there were more students involved, more abstracts published, more presentations made, and more publications produced. Also, at institutions where more faculty used a workshop on grant writing, more students were involved and there were more publications. Sabbatical use was associated with more student involvement and more publications. In addition, at institutions where more faculty had research grants, more students were involved and there were more publications. When more faculty used training for administration there were more funded grants and where more faculty had incentives to develop a new course there were more abstracts published.

**Table 5:** Rate ratio of increased productivity for institutions based on annual progress reports in 2015–2018 to participants’ use of a tool as indicated by the 2016–2017 HERI survey.

RR 95% CI	Pilots funded	No. of Students in Program	No. of Abstracts	No. of Presentations	No. of Publications	No. of Submitted grants	No. of Funded grants
**Workshop on teaching, ≥ half**	1.16 (0.81–1.69)	**1.57 (1.24–2.01)** ****	**4.04 (2.21–8.09)** ****	**1.89 (1.43–2.51)** ****	**4.75 (2.29–11.40)** ****	0.82 (0.58–1.16)	0.62 (0.35–1.13)
**Workshop on research skills, ≥ half**	0.94 (0.61–1.41)	**0.47 (0.34–0.65)** ****	0.65 (0.36–1.09)	**0.20 (0.11–0.33)** ****	**0.04 (0.00–0.24)** ****	**0.05 (0.01–0.17)** ****	**0.04 (0.00–0.18)** ****
**Workshop on grant writing, ≥ quarter**	1.43 (1.02–2.01) *	**1.49 (1.21–1.84)** ****	**0.32 (0.19–0.51)** ****	1.29 (1.03–1.62) *	**2.64 (1.64–4.34)** ****	0.64 (0.45–0.91) *	0.57 (0.28–1.09)
**Sabbatical, ≥ tenth**	1.06 (0.75–1.49)	**1.98 (1.60–2.45)** ****	**0.23 (0.13–0.39)** ****	1.23 (0.98–1.54)	**2.50 (1.56–4.09)** ****	0.64 (0.45–0.91) *	0.57 (0.28–1.09)
**Travel funds from institution, ≥ half**	0.88 (0.62–1.25)	0.76 (0.61–0.94) *	**0.22 (0.14–0.33)** ****	0.80 (0.64–1.01)	2.13 (1.22–3.93) **	0.71 (0.51–1.00)	0.73 (0.41–1.33)
**Research grants institution, ≥ half**	1.43 (1.02–2.01) *	**1.49 (1.21–1.84)** ****	**0.32 (0.19–0.51)** ****	1.29 (1.03–1.62) *	**2.64 (1.64–4.34)** ****	0.64 (0.45–0.91) *	0.57 (0.28–1.09)
**Training on administrative leadership, ≥ quarter**	1.65 (1.03–2.56) *	0.69 (0.46–1.01)	**0.07 (0.00–0.42)** ****	0.84 (0.56–1.23)	0.10 (0.00–0.55) **	1.20 (0.71–1.93)	**1.71 (0.76–3.47)** ****
**Incentive to develop new course, ≥ quarter**	0.62 (0.44–0.88) **	**0.40 (0.32–0.50)** ****	**4.33 (2.56–7.75)** ****	**0.41 (0.32–0.52)** ****	**0.42 (0.26–0.67)** ***	**0.41 (0.29–0.58)** ****	**0.34 (0.17–0.66)** ***
**Incentive to integrate technology in classroom, ≥ half**	0.72 (0.45–1.12)	**0.31 (0.21–0.45)** ****	0.65 (0.36–1.09)	**0.21 (0.12–0.34)** ****	**0.22 (0.07–0.54)** ****	**0.02 (0.00–0.07)** ****	**0.04 (0.00–0.18)** ****
**Incentive to integrate culturally competent practices in the classroom, ≥ half**	0.75 (0.51–1.10)	**0.27 (0.19–0.37)** ****	**0.37 (0.21–0.62)** ****	**0.11 (0.07–0.19)** ****	**0.27 (0.12–0.53)** ****	**0.13 (0.06–0.24)** ****	**0.04 (0.00–0.18)** ****
**BUILD grants avg ≥ $50,000**	0.98 (0.69–1.37)	**0.26 (0.20–0.34)** ****	**0.23 (0.13–0.39)** ****	**0.36 (0.27–0.47)** ****	**0.34 (0.19–0.58)** ****	0.92 (0.66–1.29)	1.60 (0.89–2.89)

Bolded indicates statistically significant after Bonferroni adjustment (P-value<0.00065)

* < .05

** < .01

*** < .001

**** < .0001

On the other hand, the associations tallied from this study suggested that fewer students were involved in projects at the institutions where more faculty used a workshop on research skills, incentives to develop a new course, incentives to integrate technology in the classroom, or incentives to integrate culturally competent practices in the classroom, and where BUILD grants were more than $50,000. Fewer abstracts were produced at the institutions where more faculty used a workshop on grant writing, sabbatical, travel funds, research grants, training on administrative leadership, or incentive to integrate culturally competent practices in the classroom, and where BUILD grants were more than $50,000. Fewer presentations were made at the institutions where more faculty used a workshop on research skills, incentive to develop a new course, incentive to integrate technology in the classroom, or incentive to integrate culturally competent practices in the classroom, and where BUILD grants were more than $50,000. There were fewer publications at the institutions where more faculty used a workshop on research skills, incentive to develop a new course, incentive to integrate technology in the classroom, or incentive to integrate culturally competent practice in the classroom, and where BUILD grants were more than $50,000. There were also fewer submitted grants at the institutions where more faculty used a workshop on research skills, incentives to develop a new course, incentives to integrate technology in the classroom, or incentives to integrate culturally competent practices in the classroom. Finally, there were fewer funded grants at the institutions where more faculty used a workshop on research skills, incentive to develop a new course, incentive to integrate technology in the classroom, or incentive to integrate culturally competent practice in the classroom.

## Discussion

In this study, we found that some of the program support tools such as workshops on teaching and grant writing skills offered at the BUILD sites had positive associations with productivity outcomes such as presentations, abstracts, and publications; however, many of the tools were negatively associated with desired faculty outcomes. This was a disappointing observation of these early outcomes and future evaluations will see if these associations hold over time. It is possible the training led to a recognition of additional time and preparation needed to develop quality research, thus leading to submission of fewer abstracts and attendance at fewer conferences, Further, the additional effort required to prepare and implement program support tools may have also led to a temporary reduction in faculty scholarly productivity, especially in a non-research intensive setting. Also, these negative association do not invalidate the importance of enhancing scientific and health education to attract students to the field. Longer terms longitudinal data will be needed to fully assess the impact of the BUILD programs.

From the data we have so far, however, one might expect that the production of abstracts would lead to more presentations and to more publications, although this expected progression is not yet apparent in these data. This may be because the data available for this analysis for BUILD phase 1 captures only two years or less of program activities in the early stages of grantsmanship where faculty are working their way toward submitting a grant by presenting and publishing their body of work but are not yet ready to submit a competitive NIH grant proposal. As additional data from annual CEC/CWEP faculty surveys becomes available, we anticipate supplementing this analysis.

We found high levels of the use of workshops on teaching, research skills, and grant writing as well as the use of travel funds and research support from their institutions. Training in administrative leadership was associated with more pilots and grants being funded, which could indicate the importance of administrative support in accomplishing research goals.

We are aware of few similar programs in undergraduate minority serving institutions. There have been similar studies of programs sponsored under the NIH Research Centers in Minority Institutions (RCMI) which were established in 1985 to support the development of biomedical research infrastructure at minority-serving institutions that had doctoral programs in health sciences and health professions [23]. Institutions that received awards from the RCMI program, some of which are also part of the DPC BUILD consortium, had positive effects on publications production and successes in supporting African American and Latino students toward doctoral degrees [23, 24], although the nature of pre-existing infrastructure to support research at such institutions may be more substantial than available in BUILD institutions. There is also evidence that grant writing workshops in particular can be successful in supporting early-career investigators from under-represented groups who are already in biomedical medical science programs, so the kinds of tools used in the BUILD consortium are feasible and can be effective if applied at the appropriate level of training [25].

Our study has limitations, such we were unable to conduct analysis of individual-level data due to as the absence of data on productivity on pilot project awardees that are specific to everyone who participated in CWEP surveys. Also, additional data on other measures of participant or institutional characteristics and faculty experience that might also influence the productivity were not available so these potential mediating effects could not be analyzed.

We treated the tools (i.e., interventions) as the same across all nine analyzed institutions, but this attempt at harmonization may have missed critical aspects of local implementation that could only be determined by a deeper qualitative approach to describe their individual characteristics. Due to a lack of a comparison group that was not exposed to the tools provided to the BUILD program so we could not assess causality. Our tests of associations that were done also relied on a small sample size of nine (the number of institutions with data) and measured the effect of the program tools without being able to make adjustment for any other factors that may affect productivity, such as the research experience of the faculty surveyed. Given these limitations, our detection of significant associations with such a small sample size even when correcting for multiple comparisons indicates that our findings are robust and that larger samples hold promise in more sophisticated analysis.

This study provides descriptive data on an important national NIH funded diversity program focused on increasing the proportions of historically underrepresented researchers in biomedical research. We found according to the HERI survey (Table 2) the program was still reaching a predominantly White group of faculty (58%) who were at more advance academic levels (54% Associate or Full Professors). However, our study, possibly reflecting the faculty composition at a career stage to conduct advanced mentoring. However, our study offers an overview of the diverse approaches that were implemented and showed the feasibility of such tools to support research among these BUILD institutions. Our study indicates that even in environments lacking opportunity, faculty have interest in learning new skills and advancing their careers. We described a range of tools, such as teaching and grant writing workshops that can be employed locally in universities serving large numbers of historically underrepresented students laying a foundation for deeper exploration of tailored program tool development in future diversity intervention research.

## Conclusions

We found a demand for tools offered in BUILD and the feasibility of such programs at more institutions serving underrepresented groups in research. To increase grant submissions and funding among underrepresented groups we need to conduct longer-term follow-up studies to capture potential lagged effects of interventions. Research careers in academia unfold over time, with publications taking months to years to be published and grants taking substantial amount of time to submit, often only after a researcher has an established publication track record. Finally, this study lays the foundation for the DPC and BUILD institutions to develop more effective interventions aimed at improving inclusion in research among faculty and students.

## Supporting information

S1 Appendix BUILD sites and their corresponding partnerships. (DOCX)
