# Supplementary material for: Enhancing grant-writing expertise in BUILD institutions: Building infrastructure leading to diversity
Source: PLoS One. 2022 Sep 22;17(9):e0274100. doi: 10.1371/journal.pone.0274100 (PMC9499285; doi:10.1371/journal.pone.0274100)
Supplement: S1 Appendix — (DOCX) [file pone.0274100.s001.docx]

**S1 Appendix. BUILD Sites and the corresponding partnerships**

**ALASKA**

**University of Alaska, Fairbanks**
BUILD BLaST: Biomedical Learning and Student Training 
PIs: Karsten Hueffer, Arleigh Reynolds
[http://alaska.edu/blast](http://alaska.edu/blast/)
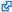

**Pipeline Partners:**
Bristol Bay Campus (CRCD Rural) (Dillingham, AK)
Chukchi Campus (CRCD Rural) (Kotzebue, AK)
Iḷisaġvik College (Barrow, AK)
Interior Aleutians Campus (CRCD Rural) (Fairbanks, AK)
Kuskokwim Campus (CRCD Rural) (Bethel, AK)
Northwest Campus (CRCD Rural) (Nome, AK)
University of Alaska Southeast (Ketchikan, Sitka, and Juneau, AK)​
**Partners:**
Alaska Pacific University (Anchorage, AK)
Diné College (Tsaile, AZ)
Fort Lewis College (Durango, CO)
Salish Kootenai College (Pablo, MT)​

**CALIFORNIA**

**California State University, Long Beach**
CSULB BUILD
PIs: Laura Kingsford, Guido G. Urizar
<http://www.csulb.edu/build>
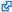

**Research Intensive Partners:**
University of California, Irvine (Irvine, CA)
University of Southern California (Los Angeles, CA)
Veterans Affairs (VA) Medical Center in Long Beach (Long Beach, CA)
**Pipeline Partners:**
Cerritos College (Norwalk, CA)
Cyprus College (Cypress, CA)
Golden West College (Huntington Beach, CA) 
Long Beach City College (Long Beach, CA)

**California State University, Northridge**
BUILD PODER: Promoting Opportunities for Diversity in Education and Research 
PIs: Crist Khachikian, Gabriela Chavira, Carrie Saetermoe, Margaret Shiffrar
<http://www.csun.edu/build-poder>
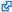

**Research Intensive Partners:**
Claremont Graduate University (Claremont, CA)
Drexel University SURF (Philadelphia, PA)
University of California, Irvine (Irvine, CA)
University of California, Los Angeles (Los Angeles, CA)
University of California, San Diego (San Diego, CA)
University of North Texas Health Science Center SMART (Fort Worth, TX)
University of Southern California, Keck School of Medicine's Bridging the Gaps Summer Research Program (Los Angeles, CA)
​**Pipeline Partners:**
East Los Angeles College (Monterey Park, CA)
Los Angeles Pierce College (Woodland Hills, CA)
Los Angeles Valley College (Valley Glen, CA)
Pasadena City College (Pasadena, CA)

**San Francisco State University**
SF BUILD: Enabling Students to Represent in Science
PIs: Leticia Márquez-Magaña (SF State), Kirsten Bibbins-Domingo (UCSF), Tung Nguyen (UCSF)
<http://sfbuild.sfsu.edu/home>
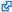

**Research Intensive Partner:**
University of California, San Francisco (UCSF) (San Francisco, CA)​

**LOUISIANA**

**Xavier University of Louisiana**
BUILD at Xavier: Project Pathways 
PIs: Maryam Foroozesh, Marguerite Giguette, Kathleen M. Morgan
[http://www.xula.edu/build](http://www.xula.edu/)
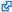

**Research Intensive Partners:**
Albert Einstein College of Medicine (New York City, NY)
Boston University School of Medicine (Boston, MA)
Dartmouth College (Hanover, NH)
Emory University (Atlanta, GA)
George Washington University (Washington, DC)
Icahn School of Medicine at Mount Sinai (New York, NY)
Johns Hopkins University (Baltimore, MD)
Louisiana State University (Baton Rouge, LA)
Louisiana State University Health Sciences Center (New Orleans, LA)
Meharry Medical College (Nashville, TN)
NYU School of Medicine (New York, NY)
Tulane University (New Orleans, LA)
Tulane University School of Medicine (New Orleans, LA)
University of California, Davis (Davis, CA)
University of California, San Francisco (San Francisco, CA)
University of Chicago (Chicago, IL)
University of Michigan (Ann Arbor, MI)
​University of Rochester (Rochester, NY)
University of Wisconsin-Madison (Madison, WI)

**MARYLAND**

**Morgan State University**
BUILD ASCEND: A Student-Centered, Entrepreneurship Development Training Model to Increase Diversity in the Biomedical Research Workforce
PIs: Farin Kamangar, Payam Sheikhattari
<http://www.morgan.edu/ASCEND>
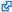

**Research Intensive Partners:**
Brown University (Providence, RI)
Howard University (Washington, DC)
Johns Hopkins Center for Health Disparities Solutions (Baltimore, MD)
Johns Hopkins Center for Health Equity (Baltimore, MD)
​Johns Hopkins University (Baltimore, MD)
Kennedy Krieger Institute (Baltimore, MD)
Lehigh University (Bethlehem, PA)
National Cancer Institute (Rockville, MD)
Northeastern University (Boston, MA)
University of Maryland Cancer Institute (Baltimore, MD)
University of Maryland, Baltimore County (Baltimore, MD)
University of Maryland, College Park (College Park, MD)
University of New Mexico Center for Participatory Research (Albuquerque, NM)
University of North Texas Health Science Center (Fort Worth, TX)
**Pipeline/Collaborator Partners:**
Baltimore City Community College (BCCC) (Baltimore, MD)
Community College of Baltimore County (CCBC) (Baltimore, MD)
JUICE/​Juxtopia, Inc. (Baltimore, MD)
Medical Education Resources Initiative for Teens (MERIT) (Baltimore, MD)
Prince George's Community College (Largo, MD) 

**University of Maryland, Baltimore County**
STEM BUILD at UMBC
PIs: Philip Rous, William LaCourse
[http://stembuild.umbc.edu](http://stembuild.umbc.edu/)
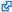

**Collaborator:**
Gallaudet University (Washington, DC)
**Pipeline Collaborators:**
Anne Arundel Community College (AACC) (Arnold, MD)
Community College of Baltimore County (CCBC) (Owings Mills, MD)
Howard Community College (HCC) (Columbia, MD)
Montgomery College (MC) (Rockville, MD)
Prince George's Community College (PGCC) (Largo, MD)
**Grad/Med:**
University of Maryland, School of Medicine (Baltimore, MD)

**MICHIGAN**

**University of Detroit Mercy**
ReBUILDetroit
PIs: Katherine Snyder
[http://rebuildetroit.org](http://rebuildetroit.org/)
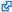

**Research Intensive Partner:**
Wayne State University (WSU) (Detroit, MI)
**Pipeline Partner:**
Henry Ford College (HFC) (Dearborn, MI)

**OREGON**

**Portland State University**
BUILD EXITO: Enhancing Cross-Disciplinary Infrastructure Training at Oregon
PI: Carlos Crespo
<https://www.pdx.edu/exito>
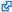

**Research Intensive Partner:**
Oregon Health and Science University (Portland, OR)
**Pipeline Partners:**
American Samoa Community College (Mesepa, Western, American Samoa)
Clackamas Community College (Oregon City, OR)
Clark College (Vancouver, WA)
Chemeketa Community College (Salem, OR)
Northern Marianas College (Susupe, Saipan, CNMI)
Oregon Department of Human Services (Oregon City, OR)
Portland Community College (Portland, OR)
University of Alaska Anchorage (Anchorage, AK)
University of Guam (Mangilao, Guam)
University of Hawaii (Honolulu, HI)

**TEXAS**

**The University of Texas at El Paso**
BUILDing SCHOLARS: Southwest Consortium of Health-Oriented Education Leaders and Research Scholars
PIs: Lourdes Echegoyen, Stephen Aley, Thomas Boland, Timothy W. Collins, Marc B. Cox, Sara Grineski, Osvaldo F. Morera (STC), Amy Wagler
[http://buildingscholars.utep.edu](http://buildingscholars.utep.edu/)
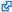

**Research Intensive Partners:**
Arizona State University (Phoenix, AZ)
Baylor College of Medicine (Houston, TX)
Clemson University (Clemson, SC)
Rice University (Houston, TX)
University of Arizona (Tucson, AZ)
University of Connecticut (Storrs, CT)
University of New Mexico (Albuquerque, NM)
University of New Mexico Health Sciences Center (Albuquerque, NM)
University of Texas at Arlington (Arlington, TX)
University of Texas at Austin (Austin, TX)
University of Texas Health Science Center at Houston (Houston, TX)
University of Texas Southwestern Medical Center (Dallas, TX)
**Pipeline Partners:**
Eastern New Mexico University (through NM-INBRE) (Portales, NM)
El Paso Community College (EPCC) (El Paso, TX)
New Mexico Highlands University (through NM-INBRE) (Las Vegas, NM)
New Mexico Institute of Mining & Technology (through NM-INBRE) (Socorro, NM)
New Mexico State University (through NM-INBRE) (Las Cruces, NM)
Northern New Mexico College (Españ​ola, NM)
San Juan College (through NM-INBRE) (Farmington, NM)
Southwestern Indian Polytechnic Institute
Texas Southern University (Houston, TX)
Transmountain Early College High School (through EPCC) (El Paso, TX)
​Western New Mexico University (Silver City, NM)
